# Crystal structures of isomeric 4-bromo-*N*-[(2-nitro­phen­yl)sulfon­yl]benzamide and 4-bromo-*N*-[(4-nitro­phen­yl)sulfon­yl]benzamide

**DOI:** 10.1107/S2056989017001578

**Published:** 2017-02-03

**Authors:** S. Naveen, A. G. Sudha, E. Suresha, N. K. Lokanath, P. A. Suchetan, M. Abdoh

**Affiliations:** a Institution of Excellence, University of Mysore, Manasagangotri, Mysuru-6, India; b Department of Chemistry, University College of Science, Tumkur University, Tumkur 572 103, India; c Department of Studies in Physics, University of Mysore, Manasagangotri, Mysuru-6, India; d Department of Physics, Science College, An-Najah National University, PO Box 7, Nablus, Palestinian Territories

**Keywords:** crystal structure, sulfonamides, N—H⋯O hydrogen bonds, C—H⋯O inter­actions, C—H⋯π inter­actions

## Abstract

The syntheses and crystal structures of the isomeric 4-bromo-*N*-[(2-nitro­phen­yl)sulfon­yl]benzamide, (I), and 4-bromo-*N*-[(4-nitro­phen­yl)sulfon­yl]benzamide, (II), are described (mol­ecular formula = C_13_H_9_BrN_2_O_5_S in each case). The asymmetric unit of (I) contains two independent mol­ecules [(I*A*) and (I*B*)], while that of (II) contains one mol­ecule. The benzoic acid aromatic ring of mol­ecule (I*A*) is disordered due to rotation about the C_ar_—C(=O) bond over two orientations in a 0.525 (9):0.475 (9) ratio. The dihedral angle between the benzene rings is 85.9 (3)° in (I*A*) and 65.22 (19)° in (I*B*), while in (II), the corresponding value is 56.7 (7)°. In the crystals of (I) and (II), N—H⋯O, C—H⋯O and C—H⋯π inter­actions generate three-dimensional networks.

## Abstract 2

The isomeric title compounds both display three-dimensional supra­molecular architectures arising from N—H⋯O, C—H⋯O, C—H⋯π and π–π inter­actions.

## Chemical context   

In recent years, *N*-(aryl­sulfon­yl)aryl­amides have received much attention as they constitute an important class of drugs for treating Alzheimer’s disease (Hasegawa & Yamamoto, 2000 ▸) and acting as anti-bacterial inhibitors of tRNA synthetases (Banwell *et al.*, 2000 ▸), antagonists for angiotensin II (Chang *et al.*, 1994 ▸) and as leukotriene D4-receptors (Musser *et al.*, 1990 ▸). Further, *N*-(aryl­sulfon­yl)-aryl­amides are known to be potent anti-tumour agents against a broad spectrum of human tumour xenografts (colon, lung, breast, ovary and prostate) in mice (Mader *et al.*, 2005 ▸). In a continuation of our work on the synthesis and crystal structures of *N*-(2-nitro­phenyl­sulfon­yl)aryl­amides (Suchetan *et al.*, 2012*a* ▸) and *N*-(4-nitro­phenyl­sulfon­yl)aryl­amides (Suchetan *et al.*, 2012*b* ▸), compounds (I) and (II) were synthesized and their crystal structures determined.
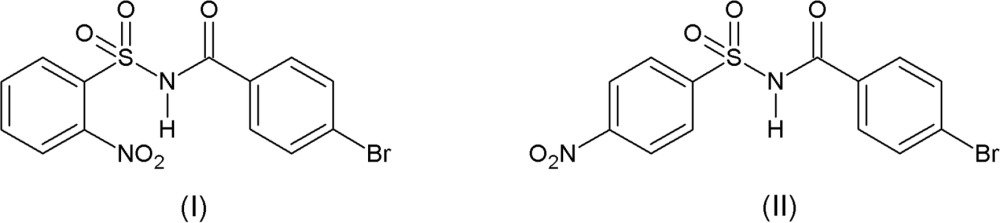


## Structural commentary   

**Figure 1:**
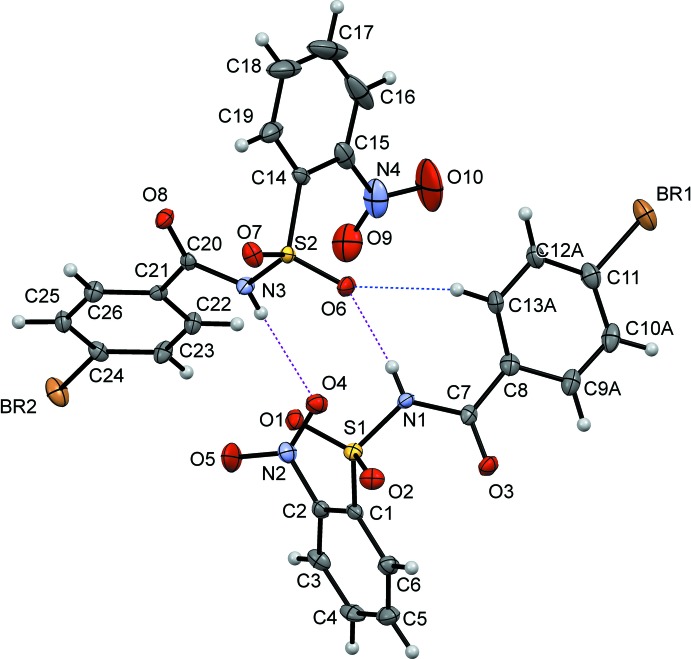
A view of (I*A*), showing displacement ellipsoids drawn at the 50% probability level.

**Figure 2:**
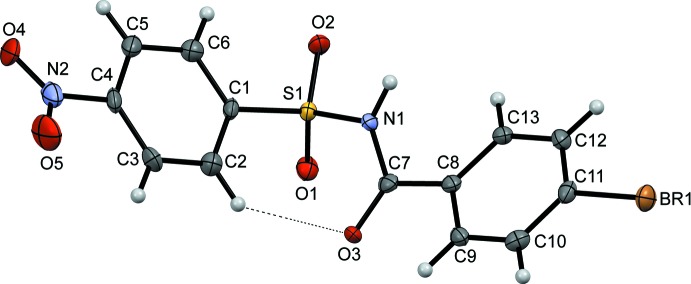
A view of (II), showing displacement ellipsoids drawn at the 50% probability level.

The asymmetric unit of (I) (Fig. 1 ▸) contains two independent mol­ecules, (I*A)* and (I*B*), while that of (II) contains one mol­ecule (Fig. 2 ▸). In both mol­ecules (I*A*) and (I*B*), the *ortho*-nitro substitution on the benzene­sulfonyl ring is *syn* to the N—H bond in the central –C—SO_2_—*N*—C(O)– segment (Fig. 1 ▸). The benzoic acid ring of mol­ecule (I*A*) is disordered due to rotation about the C_ar_—C(=O) bond over two orientations in a 0.525 (9):0.475 (9) ratio, which are inclined to each other by 45.5 (4)°. The nitro groups in both the *A* and *B* mol­ecules of (I) and the mol­ecule of (II) are twisted relative to the attached benzene­sulfonyl rings: the torsion angle C1—C2—N2—O4 in (I*A*) is 56.3 (4)°, while in (I*B*), the torsion angle C14—C15—N4—O9 is 35.6 (5)°, whereas in (II), the C5—C4—N2—O4 torsion angle has a value of 19.4 (5)°. The dihedral angle between the benzene rings is 85.9 (3)° in (I*A*), 65.22 (19)° in (I*B*) and 56.7 (7)° in (II). The conformation of (II) is supported by an intra­molecular C2—H2⋯O3 inter­action (Table 2 ▸), forming an *S*(7) motif.

## Supra­molecular features   

**Figure 3:**
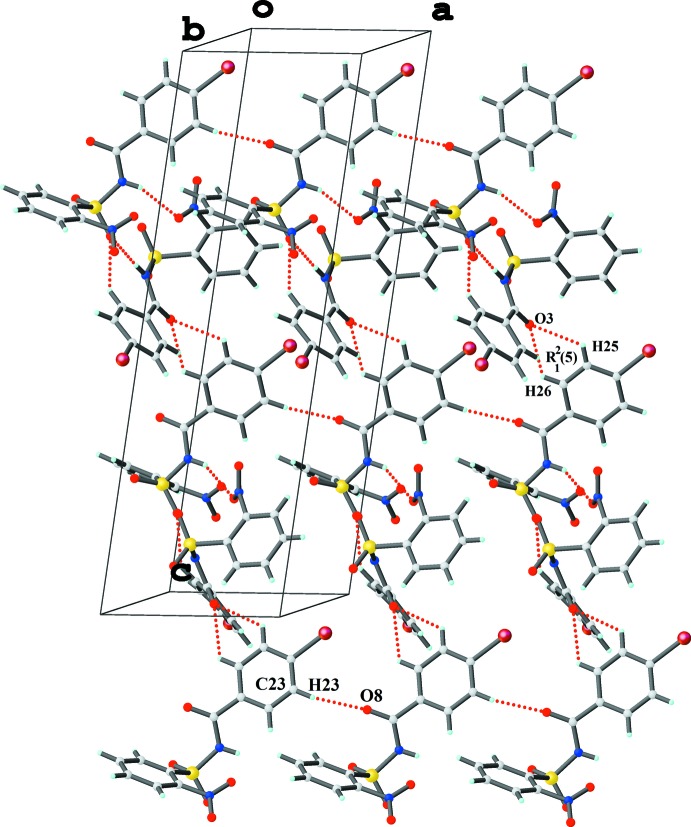
The crystal packing of (I), displaying the hetero 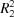
(11) dimeric supra­molecular synthon. Mol­ecules assemble along the *a* axis forming *C*(6) chains *via* C—H⋯O inter­actions while two further C—H⋯O inter­actions involving the same acceptor atom lead to the formation of an 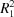
(5) network.

The crystal structure of (I) features two N—H⋯O hydrogen bonds, namely N1—H*N*1⋯O6 and N3—H*N*3⋯O4 (Table 1 ▸) between the *A* and *B* mol­ecules, resulting in a hetero dimer with graph-set motif 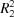
(11), which is consolidated by a C13—H13*A*⋯O6 inter­action between the *A* and *B* mol­ecules (Fig. 3 ▸). The *A* + *B* dimers assemble along the *a*-axis direction *via* C23—H23⋯O8 inter­actions, forming *C*6 chains (Table 1 ▸, Fig. 3 ▸). A dimeric 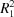
(5) network generated by the C25—H25⋯O3 and C26—H26⋯O3 inter­actions (Table 1 ▸, Fig. 3 ▸) and the 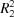
(11) network, which alternate along the *c*-axis direction, build a network of 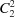
(14) and 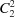
(15) chains as part of a zigzag sheet propagating in the *ac* plane, which features a short Br2⋯O3 contact [3.212 (2) Å]. Further, C10—H10*B*⋯π_1_ [where π_1_ is the nitro­benzene ring of mol­ecule (I*B*)] and C12—H12*A*⋯π_2_ [π_2_ is the bromo­benzene ring of mol­ecule (I*A*)] extend the zigzag sheets into a three-dimensional architecture, which is consolidated by several aromatic π–π stacking inter­actions [centroid–centroid separations = 3.873 (4), 3.785 (5) and 3.698 (5) Å].

**Figure 4:**
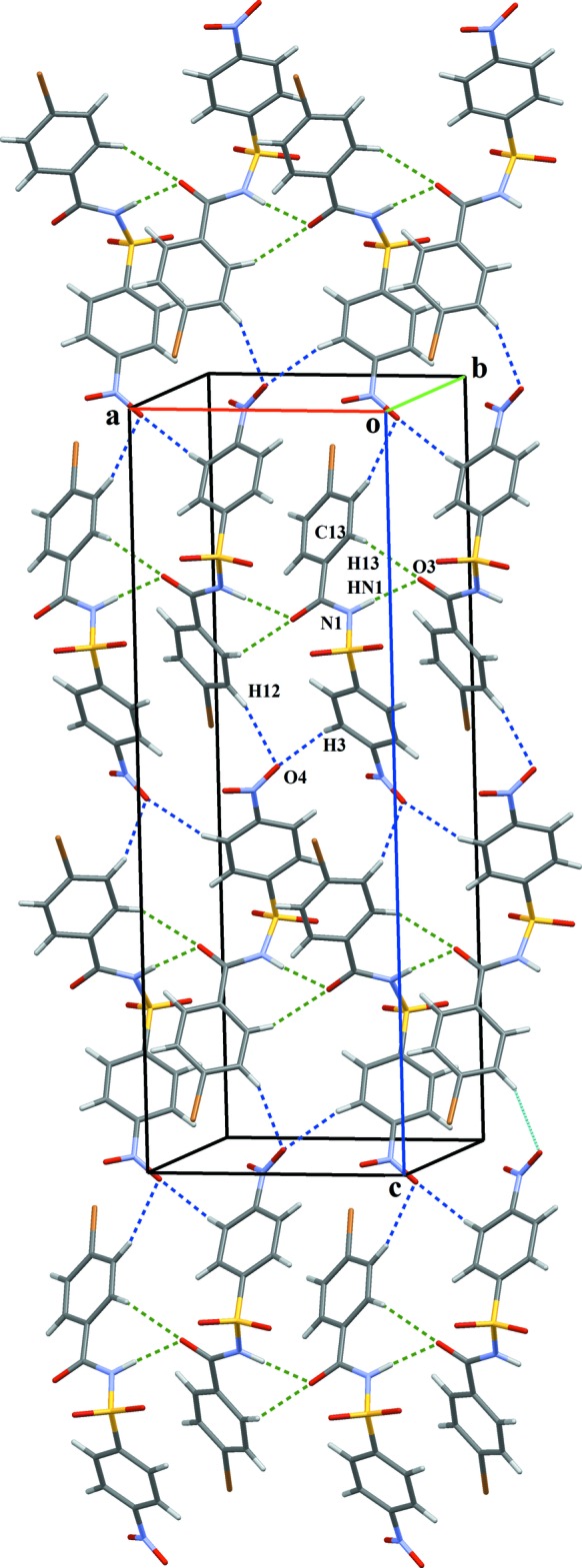
Structure-directing C—H⋯O inter­actions in the crystal structure of (II) propagating along the *b* axis as chains.

The crystal structure of (II) features N1—H*N*1⋯O3 hydrogen bonds forming *C*(4) chains along [100] (Table 2 ▸, Fig. 4 ▸): these chains are further strengthened by C13—H13⋯O3 inter­actions (Table 2 ▸) forming *C*(5) chains. The mol­ecules of neighbouring chains are inter­linked *via* C3—H3⋯O4 and C12—H12⋯O4 inter­actions (*i.e*. O4 acts as a double acceptor) and thus, a zigzag sheet propagates in the *ac* plane (Table 2 ▸). The C12—H12⋯O4 and C3—H3⋯O4 inter­actions run as *C*(13) and *C*(5) chains, respectively, along [001]. Mol­ecules in adjacent layers are linked *via* C9—H9⋯O2 and C10—H10⋯O1 inter­actions that form *C*(7) and *C*(8) chains propagating along the *b*-axis direction, and thus a three-dimensional network is obtained. A short O5⋯Br1 [3.173 (4) Å] contact is observed.

## Database survey   

A survey of the Cambridge Structural Database (Groom *et al.*, 2016 ▸) revealed 82 phenyl­sulfonyl-aryl­amide structures with different substituents attached to the benzene rings including the parent compound *N*-benzoyl­benzene­sulfonamide (Gowda *et al.*, 2009 ▸).

## Synthesis and crystallization   

Compounds (I) and (II) were prepared by refluxing a mixture of 4-bromo­benzoic acid, the corresponding substituted benzene­sulfonamide and phospho­rus oxychloride for 3 h on a water bath. The resultant mixtures were cooled and poured into ice-cold water. The solids obtained were filtered, washed thoroughly with water and then dissolved in sodium bicarbonate solutions. The compounds were later reprecipitated by acidifying the filtered solutions with dilute HCl. They were filtered, dried and recrystallized. [m.p. = 486 for (I) and 498 K for (II)]. Colourless prisms of (I) and (II) were obtained by slow evaporation of the respective solutions of the compounds in methanol (with a few added drops of water).

## Refinement details   

Crystal data, data collection and structure refinement details are summarized in Table 3 ▸. The H atoms of the NH groups in (I) and (II) were located in a difference map and later refined freely. The carbon-bound H atoms were positioned with idealized geometry and refined using a riding model with C—H = 0.95 Å, and with *U*_iso_ = 1.2*U*_eq_(parent atom).

## Supplementary Material

Crystal structure: contains datablock(s) I, II, shelx. DOI: 10.1107/S2056989017001578/hb7646sup1.cif

Structure factors: contains datablock(s) I. DOI: 10.1107/S2056989017001578/hb7646Isup2.hkl

Structure factors: contains datablock(s) II. DOI: 10.1107/S2056989017001578/hb7646IIsup3.hkl

Supporting information file. DOI: 10.1107/S2056989017001578/hb7646Isup4.cml

Supporting information file. DOI: 10.1107/S2056989017001578/hb7646IIsup5.cml

CCDC references: 1530208, 1530207

Additional supporting information:  crystallographic information; 3D view; checkCIF report

## Figures and Tables

**Table 1:** Hydrogen-bond geometry (Å, °) for (I) *Cg*1 and *Cg*2 are the centroids of the bromo­benzene ring of mol­ecule *A* and nitro­benzene ring of mol­ecule *B*, respectively.

*D*—H⋯*A*	*D*—H	H⋯*A*	*D*⋯*A*	*D*—H⋯*A*
N1—H*N*1⋯O6	0.81 (4)	2.03 (4)	2.837 (4)	172 (5)
N3—H*N*3⋯O4	0.82 (6)	2.29 (5)	3.021 (4)	148 (4)
C13*A*—H13*A*⋯O6	0.95	2.41	3.210 (8)	141
C23—H23⋯O8^i^	0.95	2.50	3.425 (4)	165
C25—H25⋯O3^ii^	0.95	2.51	3.117 (4)	122
C26—H26⋯O3^ii^	0.95	2.51	3.123 (4)	122
C12*A*—H12*A*⋯*Cg*1^iii^	0.95	2.99	3.635 (9)	126
C10*B*—H10*B*⋯*Cg*2^iii^	0.95	2.76	3.532 (8)	139

Symmetry codes: (i) 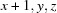; (ii) 
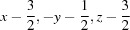
; (iii) 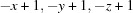.

**Table 2:** Hydrogen-bond geometry (Å, °) for (II)

*D*—H⋯*A*	*D*—H	H⋯*A*	*D*⋯*A*	*D*—H⋯*A*
N1—H*N*1⋯O3^i^	0.90	1.97	2.8530	168
C2—H2⋯O3	0.95	2.36	3.1280	138
C3—H3⋯O4^ii^	0.95	2.45	3.3199	152
C9—H9⋯O2^iii^	0.95	2.55	3.2599	132
C10—H10⋯O1^iv^	0.95	2.48	3.1081	124
C12—H12⋯O4^v^	0.95	2.56	3.4445	155
C13—H13⋯O3^i^	0.95	2.53	3.3182	141

Symmetry codes: (i) 
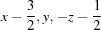
; (ii) 
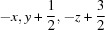
; (iii) 
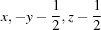
; (iv) 
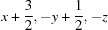
; (v) 
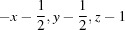
.

**Table 3:** Experimental details

	(I)	(II)
Crystal data		
Chemical formula	C_13_H_9_BrN_2_O_5_S	C_13_H_9_BrN_2_O_5_S
*M*_r_	385.19	385.19
Crystal system, space group	Monoclinic, *P*2_1_/*n*	Orthorhombic, *P* *b* *c* *a*
Temperature (K)	173	173
*a*, *b*, *c* (Å)	8.0209 (3), 14.5364 (5), 25.0008 (8)	9.6085 (4), 10.3246 (5), 27.7296 (13)
α, β, γ (°)	90, 98.499 (1), 90	90, 90, 90
*V* (Å^3^)	2882.96 (17)	2750.9 (2)
*Z*	8	8
Radiation type	Cu *K*α	Cu *K*α
μ (mm^−1^)	5.50	5.76
Crystal size (mm)	0.25 × 0.12 × 0.09	0.22 × 0.11 × 0.08

Data collection		
Diffractometer	Bruker APEXII	Bruker APEXII
Absorption correction	Multi-scan (*SADABS*; Bruker, 2009 ▸)	Multi-scan (*SADABS*; Bruker, 2009 ▸)
*T*_min_, *T*_max_	0.476, 0.610	0.491, 0.631
No. of measured, independent and observed [*I* > 2σ(*I*)] reflections	17578, 4732, 4576	12896, 2256, 2221
*R*_int_	0.051	0.055
(sin θ/λ)_max_ (Å^−1^)	0.585	0.585

Refinement		
*R*[*F* ^2^ > 2σ(*F* ^2^)], *wR*(*F* ^2^), *S*	0.049, 0.139, 1.11	0.050, 0.138, 1.12
No. of reflections	4732	2256
No. of parameters	442	203
No. of restraints	1	0
H-atom treatment	H atoms treated by a mixture of independent and constrained refinement	H atoms treated by a mixture of independent and constrained refinement
Δρ_max_, Δρ_min_ (e Å^−3^)	0.71, −1.11	1.10, −1.69

Computer programs: *APEX2*, *SAINT-Plus* and *XPREP* (Bruker, 2009 ▸), *SHELXT2014* (Sheldrick, 2015*a* ▸), *SHELXL2016* (Sheldrick, 2015*b* ▸) and *Mercury* (Macrae *et al.*, 2008 ▸).

## supplementary crystallographic information

### (I) 4-Bromo-N-[(2-nitrophenyl)sulfonyl]benzamide Crystal data

**Table:**

C_13_H_9_BrN_2_O_5_S	Prism
*M**_r_* = 385.19	*D*_x_ = 1.775 Mg m^−^^3^
Monoclinic, *P*2_1_/*n*	Melting point: 486 K
Hall symbol: -P 2yn	Cu *K*α radiation, λ = 1.54178 Å
*a* = 8.0209 (3) Å	Cell parameters from 173 reflections
*b* = 14.5364 (5) Å	θ = 4.7–64.4°
*c* = 25.0008 (8) Å	µ = 5.50 mm^−^^1^
β = 98.499 (1)°	*T* = 173 K
*V* = 2882.96 (17) Å^3^	Prism, colourless
*Z* = 8	0.25 × 0.12 × 0.09 mm
*F*(000) = 1536	

### (I) 4-Bromo-N-[(2-nitrophenyl)sulfonyl]benzamide Data collection

**Table:**

Bruker APEXII diffractometer	4576 reflections with *I* > 2σ(*I*)
Radiation source: sealed X-ray tube	*R*_int_ = 0.051
Graphite monochromator	θ_max_ = 64.4°, θ_min_ = 4.7°
phi and φ scans	*h* = −7→9
Absorption correction: multi-scan (SADABS; Bruker, 2009)	*k* = −16→16
*T*_min_ = 0.476, *T*_max_ = 0.610	*l* = −28→29
17578 measured reflections	1 standard reflections every 1 reflections
4732 independent reflections	intensity decay: 1%

### (I) 4-Bromo-N-[(2-nitrophenyl)sulfonyl]benzamide Refinement

**Table:**

Refinement on *F*^2^	Primary atom site location: structure-invariant direct methods
Least-squares matrix: full	Hydrogen site location: mixed
*R*[*F*^2^ > 2σ(*F*^2^)] = 0.049	H atoms treated by a mixture of independent and constrained refinement
*wR*(*F*^2^) = 0.139	*w* = 1/[σ^2^(*F*_o_^2^) + (0.0885*P*)^2^ + 3.8108*P*] where *P* = (*F*_o_^2^ + 2*F*_c_^2^)/3
*S* = 1.11	(Δ/σ)_max_ = 0.002
4732 reflections	Δρ_max_ = 0.71 e Å^−^^3^
442 parameters	Δρ_min_ = −1.11 e Å^−^^3^
1 restraint	

### (I) 4-Bromo-N-[(2-nitrophenyl)sulfonyl]benzamide Special details

**Table:**

Geometry. All esds (except the esd in the dihedral angle between two l.s. planes) are estimated using the full covariance matrix. The cell esds are taken into account individually in the estimation of esds in distances, angles and torsion angles; correlations between esds in cell parameters are only used when they are defined by crystal symmetry. An approximate (isotropic) treatment of cell esds is used for estimating esds involving l.s. planes.

### (I) 4-Bromo-N-[(2-nitrophenyl)sulfonyl]benzamide Fractional atomic coordinates and isotropic or equivalent isotropic displacement parameters (Å^2^)

**Table:**

	*x*	*y*	*z*	*U*_iso_*/*U*_eq_	Occ. (<1)
BR2	1.02710 (5)	0.34737 (3)	0.05488 (2)	0.02901 (17)	
BR1	0.79778 (5)	0.67350 (3)	0.56143 (2)	0.03300 (18)	
S1	0.70457 (10)	0.15258 (5)	0.40128 (3)	0.0150 (2)	
S2	0.41557 (9)	0.38557 (5)	0.30456 (3)	0.0131 (2)	
O3	0.8626 (3)	0.21365 (18)	0.50821 (9)	0.0203 (5)	
O1	0.6105 (3)	0.16337 (17)	0.34822 (9)	0.0191 (5)	
O4	0.8579 (3)	0.29764 (17)	0.32525 (9)	0.0215 (5)	
O6	0.5231 (3)	0.38082 (18)	0.35563 (9)	0.0210 (5)	
O8	0.3425 (3)	0.40157 (18)	0.18734 (9)	0.0193 (5)	
O2	0.6549 (3)	0.08544 (17)	0.43717 (10)	0.0211 (5)	
O7	0.2785 (3)	0.32341 (17)	0.29277 (10)	0.0213 (5)	
N1	0.7047 (3)	0.2553 (2)	0.42926 (11)	0.0156 (6)	
N2	0.9067 (4)	0.2238 (2)	0.31015 (11)	0.0203 (6)	
O5	0.8932 (4)	0.2001 (2)	0.26277 (11)	0.0439 (8)	
N3	0.5424 (4)	0.3737 (2)	0.25907 (11)	0.0163 (6)	
O9	0.6914 (4)	0.5303 (3)	0.31353 (14)	0.0466 (8)	
C7	0.7894 (4)	0.2739 (2)	0.48080 (12)	0.0151 (7)	
C8	0.7857 (4)	0.3714 (3)	0.49883 (14)	0.0196 (7)	
C6	1.0096 (4)	0.0750 (2)	0.43391 (14)	0.0198 (7)	
H6	0.959542	0.053680	0.463750	0.024*	
C14	0.3326 (4)	0.4980 (2)	0.29766 (12)	0.0169 (7)	
C4	1.2453 (4)	0.0791 (3)	0.38479 (15)	0.0228 (8)	
H4	1.356869	0.060691	0.381307	0.027*	
C2	0.9934 (4)	0.1604 (2)	0.35111 (14)	0.0166 (7)	
C20	0.4900 (4)	0.3876 (2)	0.20422 (12)	0.0128 (6)	
C21	0.6240 (4)	0.3802 (2)	0.16921 (12)	0.0130 (6)	
N4	0.6001 (5)	0.5792 (3)	0.33590 (15)	0.0423 (10)	
C23	0.9132 (4)	0.3909 (3)	0.15395 (13)	0.0192 (7)	
H23	1.028007	0.404991	0.166430	0.023*	
C3	1.1560 (4)	0.1341 (2)	0.34542 (15)	0.0200 (7)	
H3	1.205261	0.153578	0.315019	0.024*	
C1	0.9180 (4)	0.1309 (2)	0.39488 (13)	0.0152 (7)	
C26	0.5760 (4)	0.3523 (2)	0.11605 (14)	0.0175 (7)	
H26	0.460520	0.340667	0.103073	0.021*	
C5	1.1745 (4)	0.0505 (3)	0.42912 (15)	0.0250 (8)	
H5	1.238345	0.014104	0.456339	0.030*	
C25	0.6958 (4)	0.3415 (2)	0.08176 (14)	0.0192 (7)	
H25	0.663981	0.320850	0.045635	0.023*	
C11	0.7929 (5)	0.5504 (3)	0.53672 (15)	0.0244 (8)	
O10	0.6496 (7)	0.6324 (3)	0.37231 (18)	0.0834 (15)	
C24	0.8631 (4)	0.3613 (2)	0.10135 (13)	0.0167 (7)	
C22	0.7935 (4)	0.3995 (3)	0.18771 (13)	0.0184 (7)	
H22	0.826368	0.418684	0.224058	0.022*	
C19	0.1607 (5)	0.5033 (3)	0.27804 (14)	0.0306 (9)	
H19	0.100878	0.449361	0.265210	0.037*	
C15	0.4186 (5)	0.5781 (3)	0.31678 (14)	0.0276 (8)	
C18	0.0767 (6)	0.5865 (4)	0.27715 (19)	0.0471 (13)	
H18	−0.039147	0.590113	0.262314	0.057*	
C16	0.3325 (9)	0.6615 (3)	0.31749 (19)	0.0496 (14)	
H16	0.389190	0.715932	0.331171	0.059*	
C17	0.1592 (8)	0.6624 (4)	0.2973 (2)	0.0573 (16)	
H17	0.098513	0.718418	0.297889	0.069*	
C13A	0.6630 (9)	0.4334 (5)	0.4788 (3)	0.024 (2)	0.525 (9)
H13A	0.575449	0.414218	0.451220	0.029*	0.525 (9)
C12A	0.6632 (10)	0.5234 (5)	0.4975 (3)	0.027 (2)	0.525 (9)
H12A	0.576235	0.565144	0.483732	0.032*	0.525 (9)
C13B	0.7728 (9)	0.4457 (5)	0.4604 (3)	0.0176 (19)	0.475 (9)
H13B	0.761456	0.433511	0.422743	0.021*	0.475 (9)
C12B	0.7772 (10)	0.5349 (6)	0.4794 (3)	0.024 (2)	0.475 (9)
H12B	0.770006	0.585306	0.454956	0.028*	0.475 (9)
C9A	0.9060 (13)	0.3985 (6)	0.5443 (3)	0.0228 (18)	0.525 (9)
H9A	0.983879	0.354712	0.561645	0.027*	0.525 (9)
C10A	0.9092 (13)	0.4875 (6)	0.5630 (3)	0.0252 (18)	0.525 (9)
H10A	0.988688	0.505882	0.593063	0.030*	0.525 (9)
C9B	0.8136 (15)	0.3881 (6)	0.5514 (3)	0.021 (2)	0.475 (9)
H9B	0.829992	0.338396	0.576281	0.025*	0.475 (9)
C10B	0.8190 (15)	0.4782 (6)	0.5704 (3)	0.023 (2)	0.475 (9)
H10B	0.841876	0.488856	0.608251	0.028*	0.475 (9)
HN1	0.657 (5)	0.295 (3)	0.4102 (17)	0.015 (10)*	
HN3	0.644 (7)	0.368 (3)	0.269 (2)	0.033 (12)*	

### (I) 4-Bromo-N-[(2-nitrophenyl)sulfonyl]benzamide Atomic displacement parameters (Å^2^)

**Table:**

	*U*^11^	*U*^22^	*U*^33^	*U*^12^	*U*^13^	*U*^23^
BR2	0.0266 (3)	0.0357 (3)	0.0287 (3)	−0.00689 (15)	0.01712 (18)	−0.00913 (16)
BR1	0.0396 (3)	0.0287 (3)	0.0335 (3)	−0.01070 (17)	0.0147 (2)	−0.01360 (17)
S1	0.0117 (4)	0.0179 (5)	0.0157 (4)	0.0002 (3)	0.0030 (3)	0.0000 (3)
S2	0.0154 (4)	0.0140 (4)	0.0103 (4)	0.0014 (3)	0.0038 (3)	0.0012 (3)
O3	0.0218 (12)	0.0248 (14)	0.0140 (11)	0.0039 (10)	0.0020 (9)	0.0044 (10)
O1	0.0147 (11)	0.0253 (14)	0.0167 (12)	−0.0002 (9)	−0.0004 (9)	−0.0034 (9)
O4	0.0236 (12)	0.0209 (13)	0.0208 (12)	0.0031 (10)	0.0056 (9)	0.0024 (10)
O6	0.0236 (12)	0.0283 (14)	0.0114 (11)	0.0079 (10)	0.0035 (9)	0.0024 (10)
O8	0.0143 (12)	0.0288 (14)	0.0145 (11)	0.0032 (10)	0.0016 (9)	0.0018 (10)
O2	0.0173 (11)	0.0202 (13)	0.0271 (12)	−0.0020 (10)	0.0071 (9)	0.0032 (10)
O7	0.0247 (13)	0.0206 (13)	0.0205 (12)	−0.0071 (10)	0.0102 (10)	−0.0026 (10)
N1	0.0153 (13)	0.0183 (16)	0.0127 (12)	0.0049 (11)	0.0006 (10)	0.0028 (12)
N2	0.0232 (14)	0.0242 (17)	0.0150 (14)	0.0038 (12)	0.0075 (11)	−0.0001 (12)
O5	0.068 (2)	0.052 (2)	0.0121 (13)	0.0184 (17)	0.0063 (13)	−0.0014 (13)
N3	0.0121 (14)	0.0244 (16)	0.0131 (13)	0.0035 (11)	0.0040 (11)	0.0011 (11)
O9	0.0339 (16)	0.057 (2)	0.0490 (18)	−0.0216 (15)	0.0066 (14)	−0.0042 (17)
C7	0.0127 (14)	0.0231 (19)	0.0107 (14)	0.0001 (13)	0.0059 (12)	0.0010 (13)
C8	0.0151 (16)	0.026 (2)	0.0182 (17)	−0.0020 (14)	0.0048 (13)	−0.0019 (14)
C6	0.0222 (17)	0.0181 (18)	0.0196 (16)	0.0022 (14)	0.0044 (13)	0.0023 (13)
C14	0.0260 (17)	0.0148 (17)	0.0114 (14)	0.0053 (14)	0.0076 (12)	0.0016 (12)
C4	0.0116 (15)	0.0192 (18)	0.038 (2)	0.0015 (13)	0.0051 (14)	−0.0056 (15)
C2	0.0201 (17)	0.0133 (17)	0.0165 (16)	0.0004 (12)	0.0031 (13)	−0.0016 (12)
C20	0.0161 (16)	0.0102 (16)	0.0121 (14)	−0.0022 (12)	0.0020 (12)	−0.0001 (12)
C21	0.0123 (15)	0.0134 (16)	0.0134 (15)	0.0012 (12)	0.0023 (12)	0.0023 (12)
N4	0.057 (2)	0.037 (2)	0.0328 (19)	−0.029 (2)	0.0070 (17)	−0.0033 (17)
C23	0.0127 (15)	0.027 (2)	0.0179 (16)	−0.0034 (13)	0.0009 (12)	0.0004 (14)
C3	0.0186 (17)	0.0149 (17)	0.0284 (18)	−0.0035 (13)	0.0095 (14)	−0.0037 (14)
C1	0.0138 (15)	0.0138 (16)	0.0177 (16)	0.0016 (12)	0.0019 (12)	−0.0041 (13)
C26	0.0150 (16)	0.0211 (18)	0.0167 (16)	−0.0027 (13)	0.0031 (13)	0.0005 (13)
C5	0.0194 (17)	0.023 (2)	0.0307 (19)	0.0047 (14)	−0.0025 (14)	−0.0016 (15)
C25	0.0204 (17)	0.0231 (19)	0.0141 (16)	−0.0011 (13)	0.0026 (13)	−0.0043 (13)
C11	0.0251 (18)	0.026 (2)	0.0238 (18)	−0.0066 (15)	0.0079 (14)	−0.0058 (15)
O10	0.109 (4)	0.070 (3)	0.066 (3)	−0.047 (3)	−0.003 (3)	−0.036 (2)
C24	0.0186 (16)	0.0166 (17)	0.0171 (16)	−0.0005 (13)	0.0104 (13)	0.0008 (13)
C22	0.0168 (16)	0.0242 (19)	0.0137 (15)	−0.0028 (13)	0.0010 (12)	−0.0014 (13)
C19	0.0278 (19)	0.045 (3)	0.0198 (17)	0.0165 (18)	0.0051 (14)	0.0047 (17)
C15	0.051 (2)	0.0180 (19)	0.0166 (16)	−0.0036 (17)	0.0132 (16)	0.0021 (14)
C18	0.050 (3)	0.051 (3)	0.043 (2)	0.035 (3)	0.016 (2)	0.015 (2)
C16	0.108 (4)	0.014 (2)	0.034 (2)	−0.004 (2)	0.034 (3)	−0.0019 (17)
C17	0.085 (4)	0.041 (3)	0.054 (3)	0.042 (3)	0.035 (3)	0.023 (2)
C13A	0.027 (4)	0.025 (4)	0.018 (3)	0.002 (3)	−0.003 (3)	−0.009 (3)
C12A	0.038 (5)	0.025 (4)	0.016 (3)	0.005 (3)	0.004 (3)	−0.004 (3)
C13B	0.020 (4)	0.021 (4)	0.013 (3)	0.000 (3)	0.005 (3)	0.001 (3)
C12B	0.023 (4)	0.023 (4)	0.024 (4)	−0.005 (3)	0.001 (3)	−0.001 (3)
C9A	0.018 (4)	0.035 (5)	0.016 (3)	−0.005 (3)	0.002 (3)	−0.001 (3)
C10A	0.018 (4)	0.040 (5)	0.018 (4)	−0.009 (4)	0.003 (3)	−0.004 (3)
C9B	0.032 (6)	0.021 (4)	0.011 (4)	−0.001 (4)	0.007 (4)	−0.005 (3)
C10B	0.033 (6)	0.025 (5)	0.013 (4)	−0.004 (4)	0.009 (4)	−0.008 (3)

### (I) 4-Bromo-N-[(2-nitrophenyl)sulfonyl]benzamide Geometric parameters (Å, º)

**Table:**

BR2—C24	1.890 (3)	C4—C3	1.383 (5)
BR1—C11	1.892 (4)	C4—H4	0.9500
S1—O2	1.422 (3)	C2—C3	1.386 (5)
S1—O1	1.434 (2)	C2—C1	1.394 (5)
S1—N1	1.649 (3)	C20—C21	1.487 (4)
S1—C1	1.771 (3)	C21—C26	1.388 (5)
S2—O7	1.420 (3)	C21—C22	1.398 (5)
S2—O6	1.433 (2)	N4—O10	1.215 (5)
S2—N3	1.643 (3)	N4—C15	1.464 (6)
S2—C14	1.764 (3)	C23—C22	1.374 (5)
O3—C7	1.210 (4)	C23—C24	1.386 (5)
O4—N2	1.221 (4)	C23—H23	0.9500
O8—C20	1.213 (4)	C3—H3	0.9500
N1—C7	1.392 (4)	C26—C25	1.388 (5)
N1—HN1	0.81 (5)	C26—H26	0.9500
N2—O5	1.223 (4)	C5—H5	0.9500
N2—C2	1.473 (4)	C25—C24	1.389 (5)
N3—C20	1.389 (4)	C25—H25	0.9500
N3—HN3	0.82 (5)	C11—C10B	1.343 (10)
O9—N4	1.214 (6)	C11—C12A	1.376 (8)
C7—C8	1.489 (5)	C11—C10A	1.398 (9)
C8—C9B	1.322 (8)	C11—C12B	1.438 (8)
C8—C13A	1.373 (8)	C22—H22	0.9500
C8—C9A	1.433 (8)	C19—C18	1.383 (6)
C8—C13B	1.438 (8)	C19—H19	0.9500
C6—C5	1.392 (5)	C15—C16	1.397 (6)
C6—C1	1.393 (5)	C18—C17	1.345 (9)
C6—H6	0.9500	C18—H18	0.9500
C14—C19	1.396 (5)	C16—C17	1.407 (9)
C14—C15	1.400 (5)	C16—H16	0.9500
C4—C5	1.382 (6)	C17—H17	0.9500
			
O2—S1—O1	120.04 (15)	O9—N4—O10	124.4 (5)
O2—S1—N1	109.66 (15)	O9—N4—C15	118.8 (3)
O1—S1—N1	105.10 (14)	O10—N4—C15	116.7 (5)
O2—S1—C1	107.44 (15)	C22—C23—C24	118.7 (3)
O1—S1—C1	108.61 (15)	C22—C23—H23	120.7
N1—S1—C1	105.04 (15)	C24—C23—H23	120.7
O7—S2—O6	119.98 (15)	C4—C3—C2	118.9 (3)
O7—S2—N3	109.22 (15)	C4—C3—H3	120.6
O6—S2—N3	105.01 (14)	C2—C3—H3	120.6
O7—S2—C14	107.45 (16)	C6—C1—C2	119.0 (3)
O6—S2—C14	107.49 (15)	C6—C1—S1	117.2 (3)
N3—S2—C14	107.05 (15)	C2—C1—S1	123.6 (3)
C7—N1—S1	122.6 (2)	C21—C26—C25	120.3 (3)
C7—N1—HN1	122 (3)	C21—C26—H26	119.8
S1—N1—HN1	115 (3)	C25—C26—H26	119.8
O4—N2—O5	124.1 (3)	C4—C5—C6	120.0 (3)
O4—N2—C2	118.3 (3)	C4—C5—H5	120.0
O5—N2—C2	117.5 (3)	C6—C5—H5	120.0
C20—N3—S2	122.7 (2)	C26—C25—C24	118.7 (3)
C20—N3—HN3	117 (3)	C26—C25—H25	120.7
S2—N3—HN3	119 (3)	C24—C25—H25	120.7
O3—C7—N1	120.9 (3)	C23—C24—C25	121.9 (3)
O3—C7—C8	123.2 (3)	C23—C24—BR2	119.1 (3)
N1—C7—C8	115.9 (3)	C25—C24—BR2	119.0 (3)
C5—C6—C1	119.9 (3)	C23—C22—C21	120.8 (3)
C5—C6—H6	120.0	C23—C22—H22	119.6
C1—C6—H6	120.0	C21—C22—H22	119.6
C19—C14—C15	119.1 (4)	C18—C19—C14	120.5 (5)
C19—C14—S2	115.1 (3)	C18—C19—H19	119.8
C15—C14—S2	125.2 (3)	C14—C19—H19	119.8
C5—C4—C3	120.9 (3)	C16—C15—C14	120.4 (4)
C5—C4—H4	119.6	C16—C15—N4	117.1 (4)
C3—C4—H4	119.6	C14—C15—N4	122.4 (4)
C3—C2—C1	121.3 (3)	C17—C18—C19	120.0 (5)
C3—C2—N2	117.1 (3)	C17—C18—H18	120.0
C1—C2—N2	121.5 (3)	C19—C18—H18	120.0
O8—C20—N3	120.4 (3)	C15—C16—C17	117.8 (5)
O8—C20—C21	124.0 (3)	C15—C16—H16	121.1
N3—C20—C21	115.5 (3)	C17—C16—H16	121.1
C26—C21—C22	119.6 (3)	C18—C17—C16	122.2 (4)
C26—C21—C20	117.5 (3)	C18—C17—H17	118.9
C22—C21—C20	122.9 (3)	C16—C17—H17	118.9

### (I) 4-Bromo-N-[(2-nitrophenyl)sulfonyl]benzamide Hydrogen-bond geometry (Å, º)

Cg1 and Cg2 are the centroids of the bromobenzene ring of molecule *A* and nitrobenzene ring of molecule *B*, respectively.

**Table:**

*D*—H···*A*	*D*—H	H···*A*	*D*···*A*	*D*—H···*A*
N1—H*N*1···O6	0.81 (4)	2.03 (4)	2.837 (4)	172 (5)
N3—H*N*3···O4	0.82 (6)	2.29 (5)	3.021 (4)	148 (4)
C13*A*—H13*A*···O6	0.95	2.41	3.210 (8)	141
C23—H23···O8^i^	0.95	2.50	3.425 (4)	165
C25—H25···O3^ii^	0.95	2.51	3.117 (4)	122
C26—H26···O3^ii^	0.95	2.51	3.123 (4)	122
C12*A*—H12*A*···*Cg*1^iii^	0.95	2.99	3.635 (9)	126
C10*B*—H10*B*···*Cg*2^iii^	0.95	2.76	3.532 (8)	139

Symmetry codes: (i) *x*+1, *y*, *z*; (ii) *x*−3/2, −*y*−1/2, *z*−3/2; (iii) −*x*+1, −*y*+1, −*z*+1.

### (II) 4-Bromo-N-[(4-nitrophenyl)sulfonyl]benzamide Crystal data

**Table:**

C_13_H_9_BrN_2_O_5_S	Prism
*M**_r_* = 385.19	*D*_x_ = 1.860 Mg m^−^^3^
Orthorhombic, *P**b**c**a*	Melting point: 498 K
Hall symbol: -P 2ac 2ab	Cu *K*α radiation, λ = 1.54178 Å
*a* = 9.6085 (4) Å	Cell parameters from 195 reflections
*b* = 10.3246 (5) Å	θ = 3.2–64.4°
*c* = 27.7296 (13) Å	µ = 5.76 mm^−^^1^
*V* = 2750.9 (2) Å^3^	*T* = 173 K
*Z* = 8	Prism, colourless
*F*(000) = 1536	0.22 × 0.11 × 0.08 mm

### (II) 4-Bromo-N-[(4-nitrophenyl)sulfonyl]benzamide Data collection

**Table:**

Bruker APEXII diffractometer	2221 reflections with *I* > 2σ(*I*)
Radiation source: sealed X-ray tube	*R*_int_ = 0.055
Graphite monochromator	θ_max_ = 64.4°, θ_min_ = 3.2°
phi and φ scans	*h* = −10→11
Absorption correction: multi-scan (SADABS; Bruker, 2009)	*k* = −9→11
*T*_min_ = 0.491, *T*_max_ = 0.631	*l* = −30→32
12896 measured reflections	1 standard reflections every 1 reflections
2256 independent reflections	intensity decay: 1%

### (II) 4-Bromo-N-[(4-nitrophenyl)sulfonyl]benzamide Refinement

**Table:**

Refinement on *F*^2^	0 restraints
Least-squares matrix: full	Hydrogen site location: mixed
*R*[*F*^2^ > 2σ(*F*^2^)] = 0.050	H atoms treated by a mixture of independent and constrained refinement
*wR*(*F*^2^) = 0.138	*w* = 1/[σ^2^(*F*_o_^2^) + (0.095*P*)^2^ + 3.3998*P*] where *P* = (*F*_o_^2^ + 2*F*_c_^2^)/3
*S* = 1.12	(Δ/σ)_max_ = 0.002
2256 reflections	Δρ_max_ = 1.10 e Å^−^^3^
203 parameters	Δρ_min_ = −1.69 e Å^−^^3^

### (II) 4-Bromo-N-[(4-nitrophenyl)sulfonyl]benzamide Special details

**Table:**

Geometry. All esds (except the esd in the dihedral angle between two l.s. planes) are estimated using the full covariance matrix. The cell esds are taken into account individually in the estimation of esds in distances, angles and torsion angles; correlations between esds in cell parameters are only used when they are defined by crystal symmetry. An approximate (isotropic) treatment of cell esds is used for estimating esds involving l.s. planes.

### (II) 4-Bromo-N-[(4-nitrophenyl)sulfonyl]benzamide Fractional atomic coordinates and isotropic or equivalent isotropic displacement parameters (Å^2^)

**Table:**

	*x*	*y*	*z*	*U*_iso_*/*U*_eq_	
BR1	0.84255 (4)	0.07764 (3)	0.93310 (2)	0.0173 (2)	
S1	0.64837 (8)	0.55824 (8)	0.68971 (3)	0.0106 (3)	
O3	0.9065 (2)	0.41915 (19)	0.72287 (8)	0.0121 (5)	
O1	0.7620 (2)	0.6467 (2)	0.68874 (7)	0.0150 (5)	
O2	0.5095 (2)	0.6044 (2)	0.69698 (8)	0.0142 (5)	
O4	0.5406 (3)	0.2713 (3)	0.47917 (8)	0.0329 (7)	
O5	0.7105 (3)	0.1538 (3)	0.50614 (9)	0.0336 (7)	
N1	0.6740 (3)	0.4527 (3)	0.73423 (10)	0.0109 (6)	
N2	0.6290 (3)	0.2439 (3)	0.50936 (10)	0.0226 (7)	
C8	0.8049 (3)	0.3267 (3)	0.79323 (11)	0.0118 (6)	
C9	0.9167 (3)	0.2414 (3)	0.79975 (11)	0.0123 (6)	
H9	0.985687	0.233663	0.775389	0.015*	
C13	0.7045 (3)	0.3383 (3)	0.82959 (11)	0.0120 (6)	
H13	0.628340	0.395897	0.825434	0.014*	
C10	0.9273 (3)	0.1686 (3)	0.84136 (11)	0.0145 (6)	
H10	1.002921	0.110502	0.845651	0.017*	
C12	0.7156 (3)	0.2662 (3)	0.87166 (11)	0.0138 (7)	
H12	0.648375	0.274796	0.896577	0.017*	
C5	0.5224 (4)	0.4010 (4)	0.56487 (11)	0.0175 (8)	
H5	0.442958	0.404706	0.544537	0.021*	
C4	0.6369 (3)	0.3257 (3)	0.55290 (12)	0.0161 (7)	
C7	0.8014 (3)	0.4021 (3)	0.74789 (12)	0.0123 (7)	
C6	0.5281 (3)	0.4704 (3)	0.60744 (11)	0.0156 (7)	
H6	0.450494	0.520542	0.617544	0.019*	
C11	0.8268 (3)	0.1812 (3)	0.87667 (11)	0.0133 (7)	
C3	0.7578 (3)	0.3213 (3)	0.58033 (13)	0.0190 (7)	
H3	0.834325	0.269078	0.570736	0.023*	
C2	0.7638 (4)	0.3946 (3)	0.62190 (11)	0.0172 (7)	
H2	0.845796	0.395971	0.641024	0.021*	
C1	0.6477 (3)	0.4665 (3)	0.63527 (11)	0.0117 (7)	
HN1	0.593 (4)	0.430 (3)	0.7485 (14)	0.015 (11)*	

### (II) 4-Bromo-N-[(4-nitrophenyl)sulfonyl]benzamide Atomic displacement parameters (Å^2^)

**Table:**

	*U*^11^	*U*^22^	*U*^33^	*U*^12^	*U*^13^	*U*^23^
BR1	0.0194 (3)	0.0203 (3)	0.0123 (3)	0.00086 (11)	−0.00126 (11)	0.00536 (11)
S1	0.0107 (5)	0.0135 (5)	0.0076 (5)	0.0010 (3)	−0.0005 (2)	0.0001 (3)
O3	0.0074 (12)	0.0210 (12)	0.0079 (11)	0.0002 (8)	0.0015 (9)	−0.0005 (7)
O1	0.0172 (12)	0.0143 (11)	0.0135 (11)	−0.0032 (9)	0.0000 (8)	0.0010 (8)
O2	0.0115 (12)	0.0186 (11)	0.0125 (10)	0.0044 (9)	−0.0009 (9)	0.0006 (9)
O4	0.0218 (13)	0.0647 (19)	0.0121 (12)	−0.0082 (13)	−0.0032 (10)	−0.0123 (12)
O5	0.0352 (16)	0.0397 (16)	0.0258 (14)	0.0004 (13)	0.0069 (12)	−0.0200 (12)
N1	0.0080 (13)	0.0162 (13)	0.0085 (13)	−0.0009 (10)	0.0008 (10)	0.0037 (11)
N2	0.0187 (14)	0.0352 (17)	0.0139 (15)	−0.0089 (14)	0.0035 (12)	−0.0089 (13)
C8	0.0087 (15)	0.0155 (16)	0.0112 (15)	−0.0021 (12)	−0.0004 (12)	−0.0020 (12)
C9	0.0103 (14)	0.0161 (15)	0.0105 (14)	−0.0016 (12)	0.0014 (12)	−0.0030 (11)
C13	0.0086 (15)	0.0159 (16)	0.0115 (15)	−0.0004 (12)	−0.0005 (12)	−0.0010 (12)
C10	0.0120 (15)	0.0148 (15)	0.0168 (15)	0.0001 (12)	−0.0025 (12)	0.0002 (12)
C12	0.0126 (16)	0.0181 (16)	0.0107 (15)	−0.0023 (12)	0.0012 (12)	0.0019 (12)
C5	0.0145 (18)	0.0268 (19)	0.0112 (17)	−0.0059 (14)	−0.0010 (12)	0.0017 (12)
C4	0.0188 (16)	0.0234 (17)	0.0062 (14)	−0.0050 (13)	0.0020 (12)	−0.0016 (14)
C7	0.0097 (15)	0.0151 (16)	0.0121 (15)	−0.0019 (12)	−0.0016 (13)	−0.0043 (12)
C6	0.0134 (15)	0.0188 (16)	0.0146 (15)	−0.0002 (12)	0.0002 (12)	0.0007 (13)
C11	0.0153 (15)	0.0144 (16)	0.0102 (16)	−0.0033 (12)	−0.0029 (11)	0.0010 (12)
C3	0.0162 (17)	0.029 (2)	0.0118 (16)	0.0021 (15)	0.0044 (12)	−0.0038 (13)
C2	0.0152 (16)	0.0235 (17)	0.0130 (15)	0.0003 (13)	−0.0006 (13)	−0.0012 (13)
C1	0.0141 (16)	0.0147 (16)	0.0065 (15)	−0.0022 (11)	−0.0010 (11)	−0.0010 (13)

### (II) 4-Bromo-N-[(4-nitrophenyl)sulfonyl]benzamide Geometric parameters (Å, º)

**Table:**

BR1—C11	1.901 (3)	C13—C12	1.388 (4)
S1—O1	1.424 (2)	C13—H13	0.9500
S1—O2	1.431 (2)	C10—C11	1.381 (4)
S1—N1	1.665 (3)	C10—H10	0.9500
S1—C1	1.782 (3)	C12—C11	1.390 (4)
O3—C7	1.238 (4)	C12—H12	0.9500
O4—N2	1.226 (4)	C5—C6	1.382 (5)
O5—N2	1.219 (4)	C5—C4	1.387 (5)
N1—C7	1.384 (4)	C5—H5	0.9500
N1—HN1	0.90 (4)	C4—C3	1.389 (5)
N2—C4	1.475 (4)	C6—C1	1.384 (4)
C8—C13	1.400 (4)	C6—H6	0.9500
C8—C9	1.400 (5)	C3—C2	1.380 (5)
C8—C7	1.479 (4)	C3—H3	0.9500
C9—C10	1.381 (4)	C2—C1	1.390 (5)
C9—H9	0.9500	C2—H2	0.9500
			
O1—S1—O2	120.29 (14)	C11—C12—H12	120.6
O1—S1—N1	108.68 (14)	C6—C5—C4	117.6 (3)
O2—S1—N1	104.55 (14)	C6—C5—H5	121.2
O1—S1—C1	109.13 (14)	C4—C5—H5	121.2
O2—S1—C1	107.02 (14)	C5—C4—C3	123.4 (3)
N1—S1—C1	106.33 (16)	C5—C4—N2	118.4 (3)
C7—N1—S1	125.5 (2)	C3—C4—N2	118.2 (3)
C7—N1—HN1	123 (2)	O3—C7—N1	121.0 (3)
S1—N1—HN1	112 (2)	O3—C7—C8	122.2 (3)
O5—N2—O4	124.8 (3)	N1—C7—C8	116.8 (3)
O5—N2—C4	117.6 (3)	C5—C6—C1	119.6 (3)
O4—N2—C4	117.5 (3)	C5—C6—H6	120.2
C13—C8—C9	119.3 (3)	C1—C6—H6	120.2
C13—C8—C7	123.4 (3)	C10—C11—C12	121.7 (3)
C9—C8—C7	117.3 (3)	C10—C11—BR1	118.4 (2)
C10—C9—C8	120.4 (3)	C12—C11—BR1	119.9 (2)
C10—C9—H9	119.8	C2—C3—C4	118.3 (3)
C8—C9—H9	119.8	C2—C3—H3	120.8
C12—C13—C8	120.4 (3)	C4—C3—H3	120.8
C12—C13—H13	119.8	C3—C2—C1	118.8 (3)
C8—C13—H13	119.8	C3—C2—H2	120.6
C11—C10—C9	119.3 (3)	C1—C2—H2	120.6
C11—C10—H10	120.3	C6—C1—C2	122.2 (3)
C9—C10—H10	120.3	C6—C1—S1	117.4 (2)
C13—C12—C11	118.8 (3)	C2—C1—S1	120.4 (2)
C13—C12—H12	120.6		

### (II) 4-Bromo-N-[(4-nitrophenyl)sulfonyl]benzamide Hydrogen-bond geometry (Å, º)

**Table:**

*D*—H···*A*	*D*—H	H···*A*	*D*···*A*	*D*—H···*A*
N1—H*N*1···O3^i^	0.90	1.97	2.8530	168
C2—H2···O3	0.95	2.36	3.1280	138
C3—H3···O4^ii^	0.95	2.45	3.3199	152
C9—H9···O2^iii^	0.95	2.55	3.2599	132
C10—H10···O1^iv^	0.95	2.48	3.1081	124
C12—H12···O4^v^	0.95	2.56	3.4445	155
C13—H13···O3^i^	0.95	2.53	3.3182	141

Symmetry codes: (i) *x*−3/2, *y*, −*z*−1/2; (ii) −*x*, *y*+1/2, −*z*+3/2; (iii) *x*, −*y*−1/2, *z*−1/2; (iv) *x*+3/2, −*y*+1/2, −*z*; (v) −*x*−1/2, *y*−1/2, *z*−1.
